# Spatiotemporal Analysis and Assessment of Risk Factors in Transmission of African Swine Fever Along the Major Pig Value Chain in Lao Cai Province, Vietnam

**DOI:** 10.3389/fvets.2022.853825

**Published:** 2022-03-29

**Authors:** Hu Suk Lee, Tung Duy Dao, Le Thi Thanh Huyen, Vuong Nghia Bui, Anh Ngoc Bui, Dung Tien Ngo, Uyen Ba Pham

**Affiliations:** ^1^International Livestock Research Institute (ILRI), Animal and Human Health Program, Hanoi, Vietnam; ^2^Virology Department, National Institute of Veterinary Research, Hanoi, Vietnam; ^3^Livestock System and Environment Research Department, National Institute of Animal Science, Hanoi, Vietnam; ^4^Lao Cai Animal Husbandry and Veterinary Branch, Lao Cai, Vietnam

**Keywords:** spatiotemporal analysis, risk factors, Vietnam, value chain assessment, African swine fever (ASF)

## Abstract

African swine fever (ASF) is a contagious and lethal hemorrhagic disease with a case fatality rate approaching 100% in domestic pigs. The main objectives of this study were to describe the spatiotemporal analysis as well as to assess the potential risk factors along the pig value chain in Lao Cai province, Vietnam. A total of 925 outbreaks were reported from 2019 to 2020. The three clusters (primary, secondary and 5th) were observed near the Chinese border. The most temporal clusters were detected between May and August during the study period. In addition, we evaluated the association between ASF outbreak locations to the nearest main roads and elevation. For ASF outbreak locations to the nearest main roads, compared with the reference (<5,000 m), <1,000 m (10.22 times) and 1,000–2,000 m (1.98 times) were significantly higher occurrences of ASF. For elevation, compared to the reference (>1,500 m), the farm locations with <500 m (55.31 times) showed a significantly increased risk of ASF outbreaks. Farmers perceived that the highest risk of ASF transmission may come from collectors and slaughterers, intermediaries inside and outside the commune, feed agents and maize agents in the commune, and pig retailers. Both commercial and household pig producers considered minimizing the number of people going in and out of pig stables and improving healthcare and husbandry procedures to be both very important and feasible. There is a need for compliance by all pig producers and other actors in the pig value chain to adopt biosecurity practices. Therefore, awareness, knowledge and understanding of infection and risks of ASF need to be improved. Veterinary officials at the provincial and district levels need to improve capacity and resources to perform laboratory analysis for ASF and need to coordinate with local actors on the control and prevention of ASF in the community.

## Introduction

African swine fever (ASF) is a contagious and lethal hemorrhagic disease with a case fatality rate approaching 100% in domestic pigs (1). The disease causes huge economic losses to the pig industry and threatens food security around the world, and is classified as a notifiable disease by the World Organization for Animal Health (OIE). (2, 3). ASF has been endemic in most sub-Saharan African countries and has emerged in the Caucasus and some areas of Europe (4, 5). In Asia, the first case was reported in the northeast of China in August 2018, and then the virus was quickly spread to other countries in Asia, including Vietnam (6–8). In Vietnam, ASF outbreaks were first reported in February 2019 in Hung Yen province (250 km from the Chinese border and 50 km from Hanoi) and have rapidly spread across the entire county within a short period of time. As of Dec 2021, more than 6 million (>20% of pig production) have died and been culled across the country (7). Poor biosecurity (mainly smallholders, accounting for 65–70%) was one of the main risk factors to the rapid spread of ASF at the farm level, resulting in a huge economic impact for the pig industry in Vietnam (9, 10).

Cluster analysis is an important analytic technique in spatial or spatial-temporal epidemiology. It can detect space, time and space-time clusters of disease cases resulting from disease outbreaks. Together with remote sensing data, it is becoming popular to address the research questions in veterinary medicine (11, 12). In Vietnam, some studies have been carried out to identify the space-time clusters of Porcine reproductive and respiratory syndrome (PRRS) and Foot-and-mouth disease (FMD) (13, 14), and to estimate the spatial distribution of *Culex* mosquito abundance using remote sensing data (15). In addition, value chain assessment (VCA) is a systematic framework for describing and analyzing the inter-connected activities that transport a raw product from the producers to the final consumer (16, 17). The VCA can help us to develop cost-effective intervention strategies for the pig production system. In Vietnam, a few studies have been conducted in the livestock sector (mainly pig) (18–20).

To our knowledge, no studies have been conducted to evaluate the occurrence of ASF in space and time and to identify the potential risk factors. Therefore, the main objectives of this study were to describe the spatiotemporal analysis as well as to assess the potential risk factors along the pig value chain in Lao Cai province, Vietnam.

## Materials and Methods

### Study Area and Demonstration of Data

**Figure 1:**
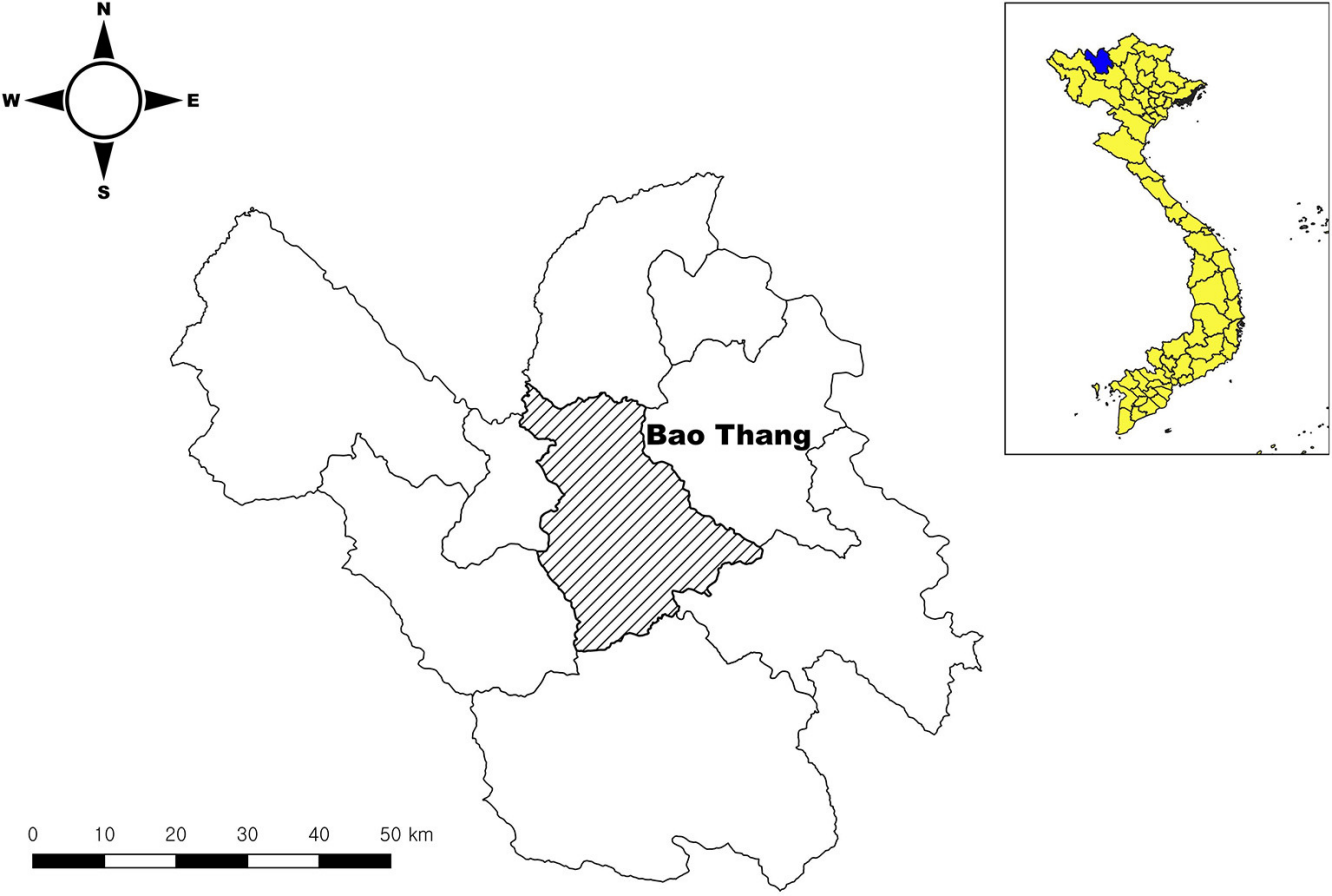
Bao Thang district where value chain assessment was conducted for our study.

Lao Cai is a highland province located in the northwest region of Vietnam, on the border with Yunnan province of China. There are estimated human and pig populations of 7,33,300 and 3,75,647 in 2019, respectively (21, 22). The annual average temperature is 23°C, and ranges between 18°C and 28°C in the mountainous region, and between 20°C and 22°C in the lowlands. The local surveillance data of ASF from 2019 to 2020 was obtained from the sub-Department of Animal Health (DAH). This surveillance data included the number of estimated cases, outbreak dates, and locations (commune, district and provincial level including GPS locations). All reported cases have been confirmed by the National Center for Veterinary Diagnostics (NCVD). The Bao Thang district, which has been highly damaged by ASF outbreaks, was selected for the value chain assessment (Figure 1). It is a lowland district with an area of 652 km^2^, and a relatively large population of 1,03,262 people in 2019. It is an important district in livestock production (especially pig and poultry) for Lao Cai province, accounting for 25–36% of the total number of pigs and over 40% of the total number of poultry.

### Data Analysis

**Figure 2:**
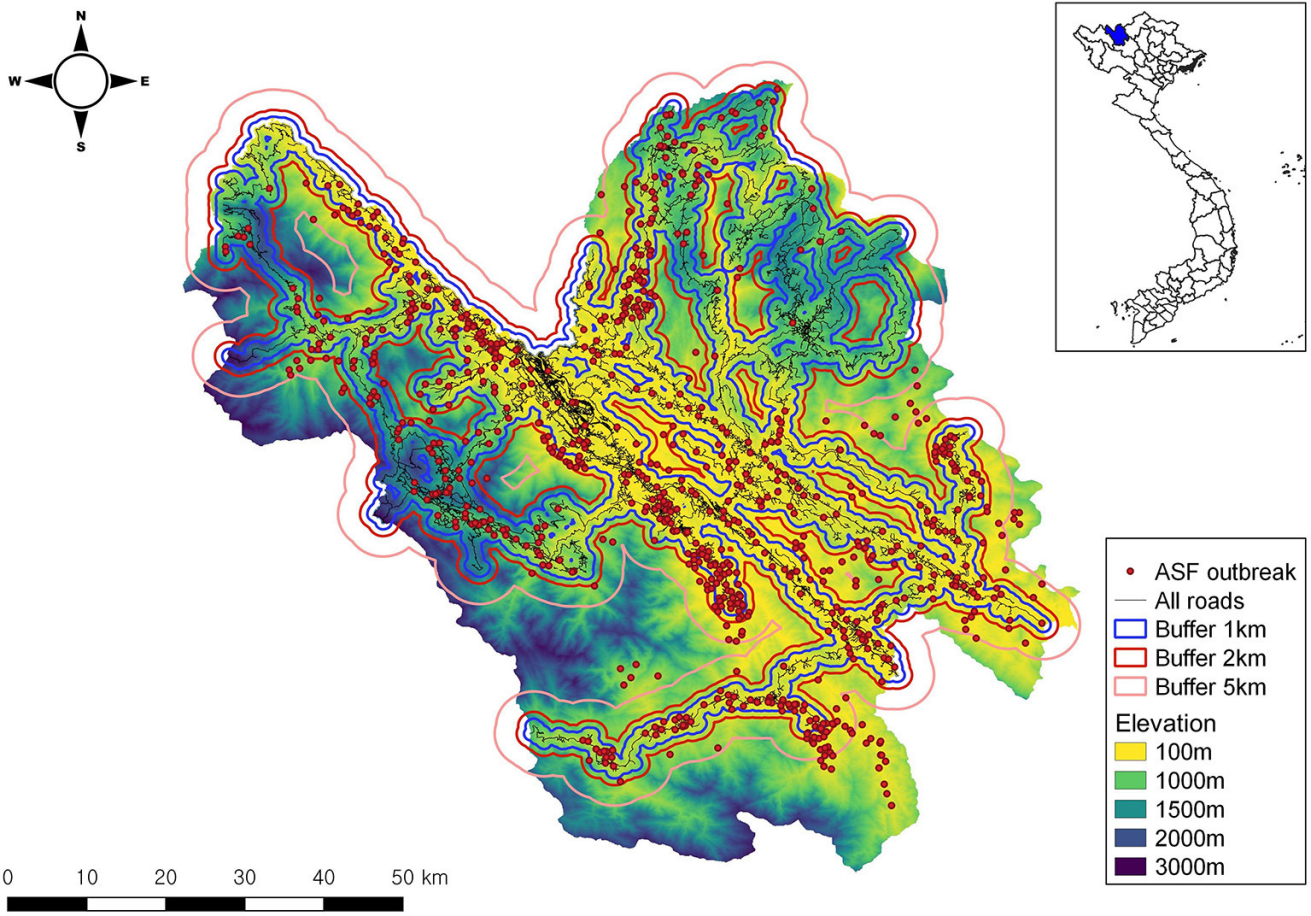
The map was created with elevation and 4 buffers by distance to the all roads in Lao Cai province.

Lao Cai province is officially divided into 17 commune-levels, and the first ASF outbreak was reported in domestic pigs in 2019. Space-time cluster analysis was carried out using the SaTScan (version 9.6 free available, http://www.satscan.org), which is commonly used to detect space and space-time clusters in public health sectors (23, 24). A space-time permutation model was selected to assess the space-time cluster occurrence as only reported cases were available, but not the pig-at-risk population data for ASF was available (24). The scan statistic executes a cylindrical window with a circular geographic base and height corresponding to time, indicating a space-time cluster. This circular window moves across each farm location and then calculates the expected cases within the window under the assumption that they are randomly dispersed in space. Clusters were identified by observed/expected cases under the null hypothesis of no clustering. For our analysis, the spatial and temporal window sizes were set to a maximum of 50%. The test statistic of the identified clusters was computed by a maximum likelihood ratio function, and the *p*-value was obtained by Monte-Carlo simulation with 999 replications of the dataset under the null hypothesis. The primary cluster can be defined as the most likely cluster, and the secondary cluster can be defined as non-overlapping clusters with less likelihood than the primary cluster. In addition, the associations between ASF outbreak locations and the nearest main road/elevation were evaluated during the study period as there have been a number of studies, which were conducted on this (25, 26). The road data were extracted from https://www.openstreetmap.org/ and transformed into shapefile. The various buffer zones (1 km, 2 km, and 5 km) were created around roads (Figure 2). The digital elevation data were obtained from NASA's Shuttle Radar Topography Mission (SRTM). The SRTM product is at a resolution of 1 arc-second (approximately 30 m). Since the outbreak locations were recorded, the number of infected farms by buffer zone along roads was calculated, and the elevation values of infected farms were extracted from the raster layer using the raster package in R (27). The number of infected farms was categorized based on distance (<1,000 m, 1,000–5,000 m, and 5,000 m>) and elevation (<500 m, 500–1,000 m, 1,000–1,500 m and >1,500 m). The negative binomial regression (NBR) model was constructed to assess the risk differences for two variables by category while <1,000 m (distance to road) and <500 m (elevation) sub-categories were used as reference, respectively. Our results were expressed as incidence rate ratio ($\frac{\text{Incidence rate in the exposed group }}{\text{Incidence rate in the unexposed group}}$) and 95% confidence interval (CI). Although Poisson models are commonly used for the analysis of count data, the number of cases showed evidence of overdispersion (variance is more than the mean) so NBR models that embraced an overdispersion term (alpha [α]) were preferred to Poisson models (28). All data were entered into Microsoft Excel and analyzed using STATA version 17.0 (StataCorp, College Station, TX). A *p*-value of less than 0.05 was considered statistically significant.

For the assessment of risk factors along the pig value chain, data collection was conducted from 10 to 24 Dec 2020 through focus group discussions (FGDs) with local authorities (e.g., livestock officers, vets and agriculture extension workers) and pig producers. FGDs are most commonly used as a qualitative method to gain an in-depth understanding of current issues. In addition, the initial findings were validated in a stakeholder feedback workshop which was organized on 13 Jan 2021. Before the interviews, the interviewees signed a consent form after either reading or listening to the text. This research was approved by the Hanoi University of Public Health Review Board (No. 186/2020/YTCC-HD3), Vietnam. All relevant questionnaires are available (Supplementary Material 1).

## Results

### Spatiotemporal Analysis

**Figure 3:**
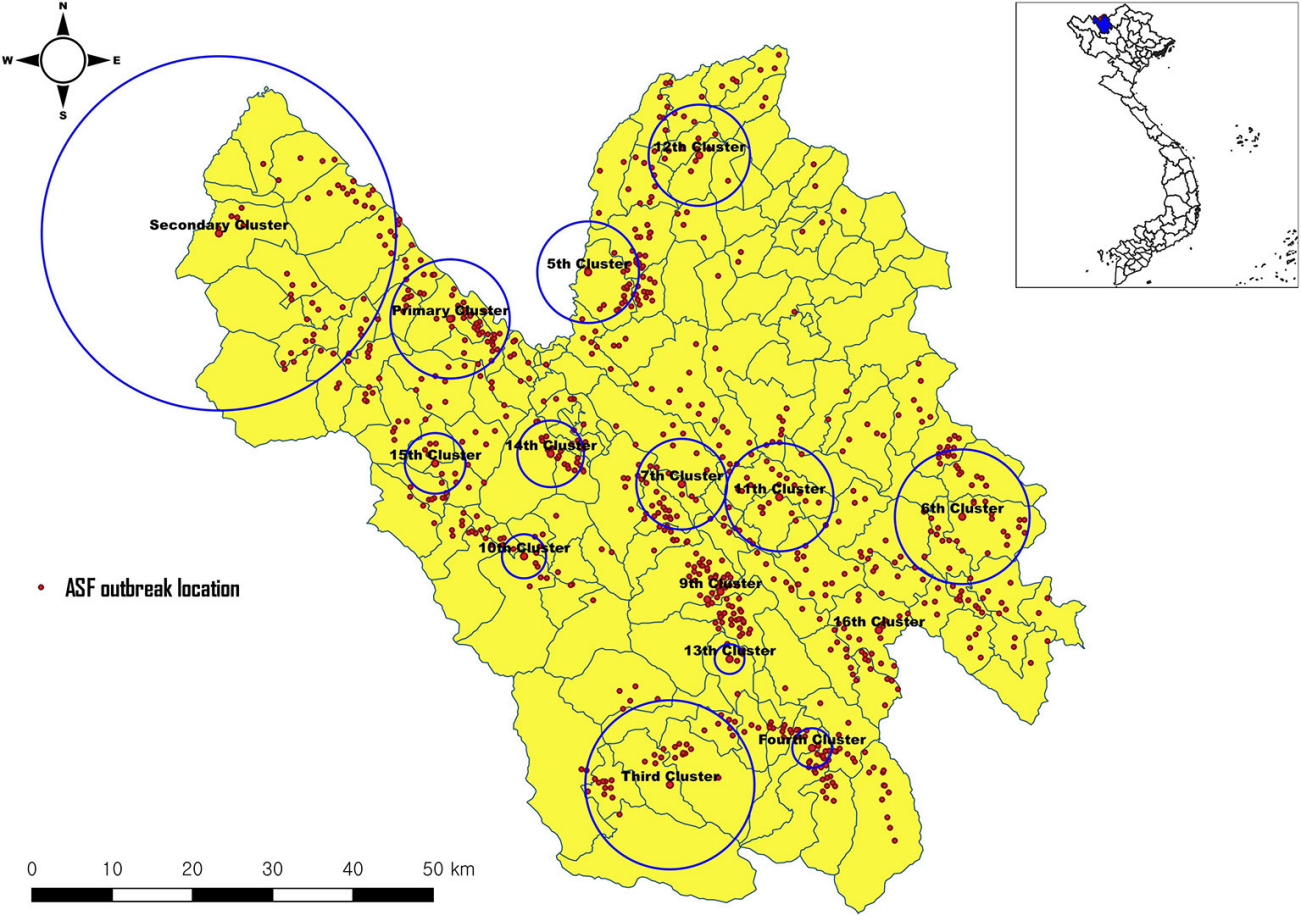
Space-time cluster analysis of ASF outbreaks from 2019 to 2020 in Lao Cai (50% at risk).

A total of 925 outbreaks were only reported in domestic pigs from 2019 to 2020. More outbreaks were reported in 2019 (708) than in 2020 (217). Using the spatial window set at 50%, a total of sixteen clusters were identified (Figure 3). The primary cluster was observed in July 2019 near the Chinese border (radius: 8.0 km), showing a ratio $\left(\frac{\text{observed cases}}{\text{expected cases}}\right)$ of 3.55 (3,618/1,019) (Table 1). The secondary cluster was identified in the northwest part of the Lao Cai province (radius: 23.78 km) with a ratio of 3.44 in July 2019. The 9th cluster showed the highest ratio (53.56), followed by the 8th cluster (25.99) and the 16th cluster (21.82). Three clusters (primary, secondary and 5th) were observed near the Chinese border. Most temporal clusters were detected between May and August during the study period. In addition, we evaluated the association between ASF outbreak locations to the nearest main roads and elevation. The NBR model showed that, compared with the reference (<5,000 m), <1,000 m (3.71 times) was significantly higher occurrences of ASF while the distance with 1,000–5,000 m was significantly preventive to the ASF outbreaks (Table 2). For elevation, compared to the reference (>1,500 m), the farm locations with <500 m (55.31 times) showed a significantly increased risk of ASF outbreaks, followed by 500–1,000 m (10.08 times) and 1,000–1,500 m (6 times), respectively.

**Table 1:** Space-time clusters of ASF from 2019 to 2020 in Vietnam (space window: 10% at risk).

**Cluster No**.	**Time year/month**	**Obs/exp = ratio**	**Radius (km)**	***P-value***
Primary	Jul/2019-Jul/2019	3,618/1,019.32=3.55	8.00	<0.001
Secondary	Jul/2019-Jul/2019	3,342/971.57=3.44	23.78	<0.001
3^rd^	Aug/2020-Dec/2020	928/64.24=14.45	11.35	<0.001
4^th^	Aug/2009-Aug/2019	1,607/279.61=5.75	2.65	<0.001
5^th^	May/2019-May/2019	724/425.75=4.05	6.82	<0.001
6^th^	Mar/2019-May/2019	461/805.22=3.06	9.04	<0.001
7^th^	June/2019-June/2019	958/149.40=6.41	6.09	<0.001
8^th^	April/2019-April/2019	355/13.66=25.99	0	<0.001
9^th^	Feb/2019-Feb/2019	224/4.18=53.56	0	<0.001
10^th^	Oct/2019-Oct/2019	326/16.26=20.04	2.96	<0.001
11^th^	April/2019-May/2019	1,305/408.17=3.20	7.27	<0.001
12^th^	Nov/2019-Jun/2020	404/36.69=11.01	6.80	<0.001
13^th^	Sep/2019-Nov/2019	489/60.70=8.06	2.00	<0.001
14^th^	Aug/2019-Aug/2019	638/130.50=4.89	4.47	<0.001
15^th^	Nov/2019-Dec/2019	142/10.68=13.30	4.07	<0.001
16^th^	Feb/2019-Feb/2019	88/4.03=21.82	3.48	<0.001

**Table 2:** Multivariable negative binomial regression (NBR) models for the distance to nearest road and elevation with incidence rate ratio (IRR) and 95% confidence interval (CI).

**Variable (n)**	**Adjusted incidence**	**95% CI rate ratios (IRRs)**
**Short distance from road**		
<1000m (579)	3.71*	2.84-4.85
1000-5000m (212)	0.69*	0.52-0.90
>5000m (151)	Ref	N/A
**Elevation**		
<500m (719)	55.31*	31.96-95.72
500-1000m (131)	10.08*	5.70-17.82
1000-1500m (78)	6.00*	3.34-10.79
>1500m (13)	Ref	N/A

The * symbol indicates statistically significant value P < 0.05.

### Assessment of Risk Factors in Transmission of ASF Along the Major Pig Value Chains

A total of three groups (7 people / group) took part in the discussion (2 groups for local authorities and 1 group for pig producers; randomly selected from a list). In general, it took 2–3 h for the FGDs.

#### Main Reasons for the Transmission of ASF as Assessed by Local Authority

In Lao Cai, the first outbreak was reported in May 2019 and then spread rapidly across the province. It was assumed that the main reasons were as follows:

- uncontrolled slaughter points in residential areas
- farmers' use of swills from restaurants and kitchens as pig feed
- keeping of livestock close to family kitchens
- people moving from households where pigs had ASF to other households
- the officials' non-compliance with anti-epidemic regulations (for example, failing to examine, take samples from and kill diseased pigs)
- direct spread through natural mating service or trading of pig semen
- the discarding of dead pig carcasses into the environment
- small households' low adoption of biosecurity practices
- announcement of ending ASF outbreak of some communes may have caused subjective psychology in disease prevention of some livestock farmers and local authorities.

In 2020, in addition to the above reasons, a number of new issues contributed to the spread of ASF. First, pig producers killed infected pigs near roads or water sources in contravention of technical procedures by not wearing biologically protective clothing and burying pigs through middlemen. Second, producers only destroyed infected pigs but not pigs in the same cage, causing diseases to spread and prolonging the outbreak. Third, the official mechanisms participating in disease prevention and control at the district and communal levels were sometimes inconsistent and erratic. Lastly, some pig producers did not report cases of ASF on their farm to officials or delayed their reports, allowing the epidemic to spread widely and making it difficult to control.

#### Perception of Risk Factors Along the Pig Supply Chain Among Pig Producers in the Study Area

**Figure 4:**
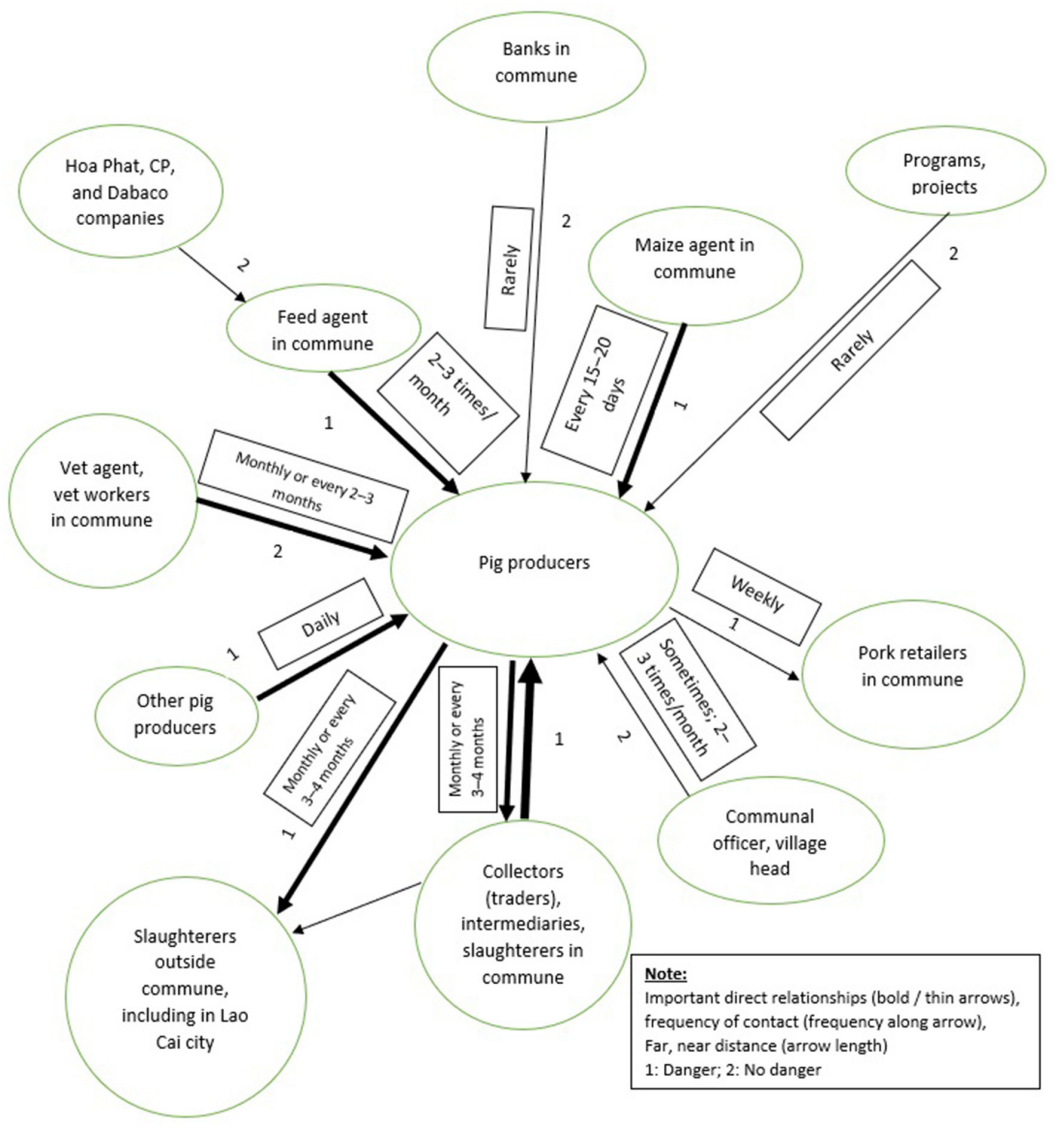
Perception*s* of danger of ASF transmission in the value chain among pig producers in the study area.

Two FGDs were held with farmers, each with different production scales. This Venn diagram (Figure 4) showed linkage of producers to other actors in the pig supply and consumption chain: Important direct relationships (bold / thin arrows), frequency of contact (write frequency along arrow), far, near distance (arrow length, inside, outside locality, province, district, commune), and assessment of the potential risk of pathogen transmission through relationships (1. Danger, 2. No danger, 3. Don't know). In general, farmers perceived that the highest risk of ASF transmission may come from collectors and slaughterers, intermediaries inside and outside the commune, feed agents and maize agents in the commune, and pig retailers. Traders bought pigs from farmers and also sold them breeding piglets. Most households bought breeding piglets from traders and did not know the origin of the pigs. In reality, traders visited many places, from household to household, and farmers. The slaughterers in the commune also visited multiple farms and different places to buy pigs; they also had to go into pig stables to catch them. An intermediary was often a trader or collector in the commune who travels to many regions to find and catch pigs. Feed and maize agents come into contact with many farmers, including those with sick pigs. However, farmers thought that the agents may spread disease, but that the risk was not as high as with traders because agents did not enter the pigpens, apart from providing breeding pigs.

## Discussion

This research was the first attempt to assess the spatiotemporal analysis of ASF using local surveillance and remote sensing data, as well as to evaluate the risk factors in the pig value chain in Lao Cai, Vietnam. It was possible that ASF cases in the local surveillance system were likely to be underestimated because small-scale farms (accounting for 65−70% of the pig population in Vietnam) lacked knowledge about livestock diseases (including ASF) and were less reachable to veterinarians and animal health workers. Moreover, all pigs must be slaughtered when a new case is confirmed on the farm, which may have resulted in reluctance for reporting.

The primary, secondary and fifth clusters were observed near the Chinese border, which might be highly associated with the illegal movements between Chinese borders as the first ASF case was reported in China (6) and rapidly spread to other countries in Asia, including Vietnam (7). It was assumed that the ASF virus was transmitted through pig movements and pork products or infected fomite (29). One study found that the strain of the ASF virus in Vietnam was the same strain as the circulating virus in China (8). In addition, it showed similar outbreak patterns of the highly pathogenic porcine reproductive and respiratory syndrome (HP-PRRS) in 2007 (30). The HP-PRRS outbreak was first detected in China, then quickly jumped in Vietnam and other Southeast Asian countries (31, 32).

Our study found that the distance to the road and elevation were reversely associated with the ASF outbreaks, which was consistent with previous studies (25, 26). Actually, unregulated slaughtering and the proximity of pig slaughterhouses to the main road is common in Vietnam (33). In addition, it is possible that farms at low elevation have better accessibility for human movement (e.g., traders and feed companies), resulting in an increased risk of ASF.

Since the first case of ASF was detected in northern Vietnam in February 2019, the number of affected provinces rapidly were increased within a short period of time (<4–5 months). Some potential risk factors have been proposed, such as illegal trading activities; low biosecurity; feeding of food scraps to pigs; not fully culling all pigs in infected farms, disposal of pig carcasses to public areas. Moreover, increased human and animal movements during the Tet holiday (Vietnamese New Year in late January or early February) may have played a significant role in spreading the ASF virus across the country.

It has been well recognized that wild boars and soft tickets can play a significant role in the transmission of the virus (34–36). In Asia, ASF in wild boars has been detected in China and South Korea (37–39). However, no studies have been implemented to evaluate the possible roles of wild boars and soft tickets for spreading a virus among farms in Vietnam. Especially, Lao Cai province is a mountainous region that may provide favorable conditions for wild boar habitats. Therefore, more research is necessary to assess the main transmission route of the ASF virus at the farm level.

Our research had some limitations in that it was likely that ASF surveillance data were underreported due to lack of awareness, animal health professionals and laboratory facilities in rural and mountainous areas. Especially, farmers were reluctant to report to the authorities because of low compensation rates and complicated/prolonged administrator procedures. In addition, it was assumed that the pig population did not rapidly change during the research period, which is very important for a space-time permutation model. Therefore, it was possible that our detected clusters may have been affected by the pig population at risk if the background population dramatically increased or decreased in one area compared to another.

During the stakeholder feedback workshop, participants discussed the risk factors for ASF transmission, especially the differences in risk levels between commercial farms and household farms. Although the risk of disease on any farm was tied to many factors, the human risk factors were lower on commercial farms because most of them applied high biosecurity procedures. In addition, they did not allow visitors, including traders, into the barn areas. However, for small-scale producers, the human movement was one of the most important risk factors as they applied fewer biosecurity practices and did not control their neighbors or traders who may move in and out of the production areas, which was consistent with a previous study (9). Mostly, livestock farmers considered producers to be high-risk actors as they visited many places, however, they rarely entered the pig stables of other farmers.

According to the interviewed pig producers, the greatest difficulties for restocking pig herds after ASF outbreaks on the farms and in the region were concerns about re-infection with ASF, high cost of breeding pigs and lack of capital for both household and commercial farms. In addition, for household farms, access to breeding pigs with known origin was also difficult. Restocking of breeding sows was slow due to difficulties in purchasing quality breeds, especially exotic breeds. Restocking fatteners was also slow due to the high price of commercial piglets. ASF outbreak reoccurred in the province in February 2020 and continued to spread across the province. However, large numbers of households still did not have a deep understanding of diseases and biosecurity. Currently, the majority of people believe that any sickness or death among pigs is caused by ASF, causing farmers to be hesitant about restocking.

Among the technical solutions discussed, both commercial and household pig producers considered minimizing the number of people going in and out of pig stables and improving healthcare and husbandry procedures to be both very important and feasible. The interviewees addressed the other most important practices were strengthening decontamination and disinfection (using lime and hormones with higher frequency), disinfection of transport vehicles and killing of mice, flies and mosquitoes. These solutions were also considered highly feasible for adoption on the ground. As for appropriate policies to overcome ASF, household farms suggested that they would expect compensation or support when their pigs are depopulated due to ASF, as well as support for purchasing disinfectants. In addition, the commercial farms expected support for purchasing disinfectants, farm materials and equipment. The priority for training needs in the context of ASF is quite similar between households and commercial farms. These include the detection and recognition of ASF and emerging diseases; understanding the risks of ASF infection and how to prevent it; training on breeds and controlled breeding practices; safe artificial insemination practices; and technical knowledge of sanitary and disease-free pig housing.

There was a high risk of ASF transmission from traders in the pig value chain, including collectors, slaughterers and retailers at the provincial, district and commune levels. These actors all participated in the sale and purchase of pigs that could be infected. Furthermore, live pigs and pork products were not subject to quarantine within the province, and their movement was not controlled. In addition, when an epidemic occurred, it took a long time to identify the disease due to the lack of resources and capacity among local animal husbandry actors and the inability to analyse samples locally for detecting the ASF virus. This allowed time for pathogens to spread through transportation and sales of sick pigs. Every year, Sub- DAH officers have been trained in disease prevention, control and surveillance. However, in-depth professional knowledge was still lacking. At the district level, some stations lack livestock and animal health specialized staff. In some districts, the specialized veterinary staff they have were sent to work other jobs. The veterinary officers at the provincial and district levels have limited resources and capacity for monitoring and surveillance of ASF. There is a need for compliance by all pig producers and other actors in the pig value chain to adopt biosecurity practices. Therefore, awareness, knowledge and understanding of infection and risks of ASF need to be improved. Veterinary officials at the provincial and district levels need to improve capacity and resources to perform laboratory analysis for ASF and need to coordinate with local actors on the control and prevention of ASF in the community.

## Data Availability Statement

The raw data supporting the conclusions of this article will be made available by the authors, without undue reservation.

## Ethics Statement

The studies involving human participants were reviewed and approved by Hanoi University of Public Health. The patients/participants provided their written informed consent to participate in this study. The animal study was reviewed and approved by Hanoi University of Public Health.

## Author Contributions

HL designed and ran the statistical analyses, wrote the draft of the manuscript, and prepared all the figures and tables presented. TD and LH collected the data. All authors contributed to the conception and design of this study and editing and comprehensive revision of this manuscript.

## Funding

This study was funded by the CGIAR Research Program on Livestock.

## Conflict of Interest

The authors declare that the research was conducted in the absence of any commercial or financial relationships that could be construed as a potential conflict of interest.

## Publisher's Note

All claims expressed in this article are solely those of the authors and do not necessarily represent those of their affiliated organizations, or those of the publisher, the editors and the reviewers. Any product that may be evaluated in this article, or claim that may be made by its manufacturer, is not guaranteed or endorsed by the publisher.
