# Supplementary material for: Spatiotemporal Analysis and Assessment of Risk Factors in Transmission of African Swine Fever Along the Major Pig Value Chain in Lao Cai Province, Vietnam
Source: Front Vet Sci. 2022 Mar 29;9:853825. doi: 10.3389/fvets.2022.853825 (PMC9002011; doi:10.3389/fvets.2022.853825)
Supplement: Supplementary file 1 [file Table_1.DOCX]

## ***Focus Group Discussions (FGDs) with local livestock officers***

**CONTENTS OF THE GROUP DISSCUSSION WITH VETERINARY AND AGRICULTURAL EXTENSION OFFICERS**

**Social provincial districts:**

**Time:**

**Coordinator:** ………………………… **Recorder:**

**List of FGD participants (name, location, work address)**

**CURRENT SITUATION OF ASF IN LOCAL (PROVINCE / DISTRICT)**

**2.1. How does the degree of African swine fever disease (ASF) impact farmers' livelihoods?**

| **Type of pig production** | **Loss of pigs by ASF** | | **Level of the impact to livelihood of the producer (scoring from 1 to 5 according to the importance of the impact)** | **Note** |
| --- | --- | --- | --- | --- |
|  | **Percentage of farms having pig got ASF** | **Average pig died by ASF/ farm (min-max)** |  |  |
| Breeding large farm/ company |  |  |  |  |
| Fattening large farm/ company |  |  |  |  |
| Fattening medium farm |  |  |  |  |
| Fattening small farm |  |  |  |  |
| Household raising breeding sows |  |  |  |  |
| Household raising fatteners |  |  |  |  |
| Cooperative |  |  |  |  |

**2.2. What factors are considered to be the risk of infection with African swine fever disease (ASF) in the locality?**

**2.3. (Provide information on what resources, knowledge and practices are required to prevent ASF transmission in immediate future)**

|  | **Current status** | **Difficulties, challenges, and gaps** | **Advantages, potential and expectation** | **Solutions short and long term** |
| --- | --- | --- | --- | --- |
| Resource |  |  |  |  |
| Knowledge |  |  |  |  |
| Practices and actions |  |  |  |  |

**2.4. (Provide a brief assessment on the diagnostic and surveillance capacity of the Lao Cai province to detect, prevent and control ASF)?**

**2.5. Are policies, programs and projects related to the control of ASF in the locality?**

Current:

Gaps, difficulties and challenges

## ***Focus Group Discussions (FGDs) with pig producers***

**FGDS WITH FARMERS**

**Commune:** …………………..**District:**………………….. **Province:**

**Time:**

**Facilitator:**………………….. **Note taker**:

***Two groups of 7–8 pig producers (one men and one women group) of different production scales discussing linkages in pig production, supply and consumption chains to identify the factors and risk of transmission African swine fever disease (AFS) along the value chain***

Participants

2.1. Mapping the main local pig supply chains, ration of product chains: From producer to consumer

2.2. Venn mapping linking producers to other actors in the pig supply and consumption chain: Important direct relationships (bold / thin arrows), frequency of contact (write frequency along the arrow), far, near distance (arrow length, inside, outside locality, province, district, commune); assess the risk of pathogen transmission through relationships (1. Danger, 2. No danger, 3. Don't know), explain?
